# Analysis of an Article in the American Journal of the Medical Sciences, on Hanging

**Published:** 1840

**Authors:** 


Art. XIV.—*Report of a Series of Experiments made by the Medical Faculty of Lancaster, upon the body of Henry Cobler Moselman, executed in the Jail Yard of Lancaster Co., Penn. on the* 20*th December*, 1839. Amer. Jour. of the Med. Sciences May 1840.

This is the title of the first article in 51st No. of the American Journal of the Medical Sciences. It was prepared by a committee consisting of Doctors W. L. Atlee and Wm. B. Fahnestock, appointed at a meeting of the physicians of Lancaster, held four days subsequent to the execution, for the purpose of comparing the notes of the different gentlemen who recorded the results of the experiments.

In addition to these, there were present, and assisting in the experiments, several physicians from Chester and Lancaster counties, the physicians and medical students of the town of Lancaster, and Dr. J. K. Mitchell, Professor of Chemistry in the Philadelphia Medical Institute, and W. R. Johnston, Professor of Chemistry in the Medical Department of Pennsylvania College.

Permission having been obtained from the Sheriff of the county, some days before the execution, to make any experiments, not in direct violation of law—these gantlemen, in order to prevent confusion, regularly organized themselves into a body—appointed persons to attend to specified duties, and arranged a programme to guide them in their operations. They obtained from the Penn. Med. College, a galvanic battery, "composed of two hundred pairs of Wollaston's plates, and constructed on Prof. Hare's plan of double trough and lever." From the Lancaster Conservatory of Arts and Sciences, an electrical machine, plate two feet in diameter, and a battery composed of "three very large jars."

Thus judicious and very ample preparations were made for a course of experiments, the most important results of which we shall proceed to state, beginning with the Galvanic.

At forty-seven and a half minutes after execution, the positive pole of the battery being placed upon the left side of the neck, and the negative under the left seventh rib, and the fluid being thrown upon the plates, there followed "a spasmodic action of the muscles, supplied by the respiratory nerves." At forty-eight minutes after execution, the positive pole being retained upon the neck, the negative was applied to the epigastrium, "breaking the circuit frequently by patting the skin with the pole. This produced a violent action of the pectoral muscles, and established the respiratory action, producing audible sounds of breathing, with corresponding motions of the mouth, opening and closing regularly."—"Fifty minutes after execution, the positive pole being retained, the negative was passed along the linea alba down to the pubis. As the pole descended, the respiratory action became stronger and stronger, and when below the umbilicus, it became very powerful, particularly the expiratory efforts. The action of the respiratory organs was general, and air passed in and out of the lungs regularly. The mouth being closed, and a lighted candle held to the nose, the flame was blown out with force. This was repeated five times in quick succession. The candle could not be extinguished so long as the pole was applied above the umbilicus, but so soon as the skin was patted below the umbilicus, these marked results took place. There were regular inspirations and expirations, the flame of the candle passing in and out, so as to singe the hair in the nostrils."—Fifty-seven minutes after execution, the positive pole being retained, the negative was removed to the anterior part of the left thigh. Applied to the upper third of the thigh, the same phenomena occurred," (as those just detailed,) "accompanied with still stronger expiration; but below that point the respiratory muscles did not act well."

The power of the battery was now varied by detaching the pole at the negative end of the battery, and carrying it backwards and forwards along the plates. "It was ascertained that the muscular contractions commenced at the 23d pair of plates, and that as the force of the battery was increased, the action of the muscles became stronger."

One hour and five or ten minutes after execution, the whole power of the battery being again employed, and the positive pole being placed upon the left side of the neck, and the negative shifted successively from the left to the right aliac region—to the glutæus maximus over the sciatic nerve—to the internal surface of the thigh—and to the left knee—there were produced in the same order of succession, contraction of the muscles of the left thigh—of the right thigh—of the gluteal and other muscles, throwing the thigh outwards—slight contraction of the adductors—and finally of those upon the anterior part of the thigh. One hour and twenty minutes after execution, the positive pole being placed over the supra orbital nerve, and the negative on the left side of the chest, "all the muscles of the face were thrown into violent action; the angles of the mouth were drawn up, the eyelids closed with a tremulous motion; and the occipito-frontalis was drawn and put into action, giving motion to the scalp. In the contraction of the muscles of the face, in no instance was there an expression of passion, but merely a distortion of the countenance, which Professor Mitchell significantly termed "grimace." The positive pole being retained, and the negative shifted to various parts of the arm and fore-arm, produced various movements of the arm, and flexion and extension of the fingers, according as it was moved from place to place. Being afterwards applied to different parts of the face, various contractions were produced, particularly, strong compression and closing of the mouth. When applied to the nose, the muscles of the face generally acted.

One hour and thirty minutes after execution, the positive pole being placed on the back of the neck, and the negative carried down the spine—produced contraction of the muscles of the back—being placed upon the gluteal, the leg was thrown outwards—upon the posterior part of the thigh, the foot was raised and the leg flexed upon the thigh, &c. The negative pole being carried to the forehead and different parts of the face, " those muscles of the face used in mastication were thrown into action, and simulated chewing. There was also a grinding motion of the jaw, and a motion of the lips as in tasting."

GALVANIC EXPERIMENTS AFTER EXPOSING VARIOUS NERVES BY DISSECTION.

About one hour and thirty-five minutes after execution, the negative pole being placed upon the epigastrium, and the positive on the left par vagum, insulated, there "followed slight contraction of the muscles of the face, of the side, and of the intercostals." The descendens noni and spinal accessory of the right side being now exposed, the positive pole was placed upon each in succession, and the negative upon the epigastrium, but without any results.

At one hour and thity-seven minutes after execution, the positive pole being placed upon the right supra-orbital nerve exposed on the forehead, but not insulated, there followed slight contraction of the muscles of the right side of the face, and stronger contraction of the masseters. The positive pole being placed upon the inner side of the integuments inverted upon the forehead, there was slight contraction of the muscles of the face ; but stronger when the pole was placed upon the cuticular surface of the integuments restored. "The par vagum on each side of the neck, being divided, the positive pole applied to the forehead, and the negative to the epigastrium, the results were the same as before the division." The negative pole being retained upon the epigastrium, and the positive applied to the cut end of the lower portion of the divided par vagum of the right side, "there was slight contraction of the muscles of the right side of the face.

From these last experiments, and afterwards making some, with the poles applied to the skin, it was found that nothing was gained by exposing the nerves, and the dissection was not prosecuted as far as had been arranged in the programme.

About one hour and forty-three minutes after execution, the spinal marrow being divided between the third and fourth cervical vertebræ—the positive pole placed upon the divided ends, and the negative upon the sciatic nerve, (the pole being also in contact with divided fibres of the muscle,) contraction of the gluteus maximus followed. The negative being shifted to the skin of the muscles, it was followed by stronger contractions. Being again shifted to the sciatic nerve insulated upon the handle of a knife "the effects were not so strong, and were more local."

One hour and fifty-nine minutes after execution, "the positive pole being placed in the incision on the neck, and the negative upon the external surface of the pericardium, the muscles of the face moved, and the eyes opened and shut repeatedly. No action of the heart." The positive pole being placed upon the skin of the neck, the same results followed, but in a more striking degree.

At two hours and sixteen minutes after execution, the positive pole being placed upon the right side of the face, and the negative upon the external surface of the heart, there was some motion about the face, but no action of the heart. The positive pole being retained, and the negative passed into the right and left ventricles, through punctures in the pulmonary artery and aorta, " there followed a vermicular motion of the periphery of the right auricle."

About two and a half hours after execution, some few experiments were performed with the electrical battery, by which some slight muscular contractions were produced, but no very striking results.

Experiments and observations were made on the motions of the heart after respiration was suspended, and the quantity of the air in the lungs after death. The following table presents the results of those made on the pulse and the action of the heart.

*Dr. Burrowe's Report on the Pulse.*

| | | | | | | | |
|---|---|---|---|---|---|---|---|
| 3 | minutes | after respiration | was suspended, | pulse | 144 | in a | minute. |
| 3½ | " | " | " | " | 120 | " | " |
| 4 | " | " | " | " | 120 | " | " |
| 5 | " | " | " | " | 150 | " | " |
| 6 | " | " | " | " | 150 | and scarcely preceptible. | |
| 6⅓ | " | " | " | " | 155 | " | " |
| 7 | " | " | " | " | 155 | " | " |
| 8 | " | " | " | " | imperceptible. | | |
| 8½ | " | " | " | no pulse at the wrist. | | | |

*Prof. Mitchell's Report on the Heart and Lungs.*

4 minutes after tightening the cord sound of the heart obscure, rythm perfect,

4½ minutes after tightening the cord, sound of the heart less confused,

5 " pulsations so frequent that they cannot be counted,

5½ " sounds scarcely audible, pulsations very frequent,

| | | |
|---|---|---|
| 7 | " | pulsations 120 in a minute, |
| 7½ | " | pulsations 132 in a minute. |
| 10 | " | " 60 " " |
| 10¼ | " | more sound than percussion of the heart, |
| 10½ | " | pulsations 60 in a minute, |
| 11 | " | distinct as to sound, and no percussion, |
| 12 | " | pulsations 54 in a minute, |
| 12¾ | " | nothing audible, |
| 13 | " | sound entirely gone, |

CHEMICAL ANALYSIS OF THE BREATH.

The day before the execution, 16 ounces, in bulk, of expired air were collected, and carefully secured in bottles well corked and sealed. Three minutes after execution, and before the noose around the neck was loosened, the trachea was perforated by means of a trochar, and there was collected 12 ounces in bulk of expressed air. A portion was analysed by Prof. Johnston of Philadelphia, and the remainder by Doctor Atlee of Lancaster. Prof. Johnston taking a portion of that obtained before execution, and having properly desiccated it, subjected it to the action of pure potassa, by which 2,609 per cent. was absorbed. Taking the remainder thus freed from carbonic acid, and detonating it with hydrogen, he obtained 17,84 per cent. "of the original bulk of air before the carbonic acid had been separated" of oxygen. This then would necessarily leave 79,551 per cent. for nitrogen. Having subjected that obtained after execution also to the action of pure potassa, 7,7 per cent. of carbonic was absorbed. In attempting to produce detonation of the remainder with hydrogen, to test the presence of oxygen, he was unsuccessful in every effort, notwithstanding the proportions of the air and hydrogen were greatly varied. He afterwards heated phosphorus in it to fusion, without any combustion ensuing. It was then subjected to the action of peroxide of nitrogen, but with the same want of success as to the detection of the presence of oxygen. From this it would appear that in 100 parts of the respired air—

| | | |
|---|---|---|
| Before execution there was— | Oxygen | 17 84 |
| | Nitrogen | 79 551 |
| | Carbonic Acid | 2 609 |
| | | 100 00 |

| | | |
|---|---|---|
| After execution there was— | Carbonic Acid | 7 7 |
| | Nitrogen | 92 3 |
| | Oxygen | 0 0 |
| | | 100 00 |

Dr. Atlee in his analysis subjected the air obtained before execution, first to the action of phosphorus, to ascertain the quantity of oxygen; he found 14,80168 per cent. Subjecting the remainder to the action of alcoholic potassa he found 31,22213 per cent. of carbonic acid—which would leave 82,076107 for nitrogen. In the air obtained after execution he found by means of alcoholic potassa, 6,82214 per cent. of carbonic acid. Taking the remainder thus freed from carbonic acid and putting a piece of pure potassium in it, he found part of it converted into a protoxide. A piece of phosphorus was then fused in the same, and a thin white vapour was emitted. Believing from this that there was a trace of oxygen in it, he took another portion and detonated it with hydrogen; by which he detected the presence of 1,06944 per cent.

According to Dr. Atlee's analysis then, there were in 100 parts of respired air—

| | | |
|---|---|---|
| Before execution— | Oxygen | 14 80168 |
| | Carbonic Acid | 3 122213 |
| | Nitrogen | 82 076107 |
| | | 100 |
| After execution— | Oxygen | 1 06944 |
| | Carbonic Acid | 6 82214 |
| | Nitrogen | 92 10842 |
| | | 100 |

A *post mortem* examination of the body was made two hours after execution, when there was found an enlarged and excessively congested state of the liver. "A piece of it being taken into the hand and squeezed, blood passed out as from a saturated sponge." Its convex surface presented a "marbled appearance." The spleen also was congested—the intestines "somewhat injected"—stomach healthy. "The jugular vein having been accidentally cut while seeking for the par vagum, a large quantity of blood was discharged from it." "Immediately after dividing the spinal marrow, there ran out about four ounces of serous fluid, followed by an immense discharge of blood which continued to flow for a considerable time after." There was no dislocation of the cervical vertebræ—no rupture of the transverse ligament—no fracture of the processus dentatus. A phrenological examination was made, but as this relates to the crime for which the execution was ordered, and not to the effects of that execution, we shall pass it by

